# Decoding of miR-7-5p in Colony Forming Unit–Hill Colonies as a Biomarker of Subclinical Cardiovascular Disease—A MERIT Study †

**DOI:** 10.3390/ijms241511977

**Published:** 2023-07-26

**Authors:** Sherin Bakhashab, Hamzah Pratama Megantara, Dimas Kirana Mahaputra, Josie O’Neill, Jason Phowira, Jolanta U. Weaver

**Affiliations:** 1 Biochemistry Department, King Abdulaziz University, Jeddah 21589, Saudi Arabia; sbakhashab@kau.edu.sa; 2 Translational and Clinical Research Institute, Newcastle University, Newcastle upon Tyne NE2 4HH, UK; hamzahpratama10@gmail.com (H.P.M.); dimaskirana11@gmail.com (D.K.M.); j.o'neill4@newcastle.ac.uk (J.O.); jphowira@gmail.com (J.P.); 3 Center of Excellence in Genomic Medicine Research, King Abdulaziz University, Jeddah 2189, Saudi Arabia; 4 Faculty of Medicine, Universitas Indonesia, Jakarta 10430, Indonesia; 5 Department of Diabetes, Queen Elizabeth Hospital, Gateshead, Newcastle Upon Tyne NE9 6SH, UK; 6 Vascular Biology and Medicine Theme, Newcastle University, Newcastle upon Tyne NE1 7RU, UK

**Keywords:** cardiovascular disease, colony forming unit–Hill, miR-7-5p, inflammation, proangiogenic cells, type 1 diabetes mellitus

## Abstract

Colony forming unit–Hill (CFU–Hill) colonies were established to serve as a sensitive biomarker for vascular health. In animals, the overexpression of miR-7-5p was shown to be pro-atherogenic and associated with increased cardiovascular disease (CVD) risk. In a MERIT study, we aimed to explore the role of miR-7-5p expression in CFU–Hill colonies in type 1 diabetes mellitus (T1DM) and the effect of metformin in subclinical CVD. The expression of miR-7-5p in CFU–Hill colonies in 29 T1DM subjects without CVD and 20 healthy controls (HC) was measured. Metformin was administered to T1DM subjects for eight weeks. MiR-7-5p was upregulated in T1DM whereas metformin reduced it to HC levels. MiR-7-5p was positively correlated with c-reactive protein, and C-X-C motif chemokine ligand 10. The receiver operating characteristic curve revealed miR-7-5p as a biomarker of CVD, and upregulated miR-7-5p, defining subclinical CVD at a HbA1c level of 44.3 mmol/mol. Ingenuity pathway analysis predicted miR-7-5p to inhibit the mRNA expression of Krüppel-like factor 4, epidermal growth factor receptor, insulin-like growth factor 1 receptor, v-raf-1 murine leukemia viral oncogene homolog 1 and insulin receptor substrate ½, and insulin receptor, while metformin activated these miRNAs via transforming growth factor-β1 and Smad2/3. We proved the pro-atherogenic effect of miR-7-5p that maybe used as a prognostic biomarker.

## 1. Introduction

Cardiovascular disease (CVD) remains the leading cause of death and rising healthcare expenditure globally, with an estimated mortality of 20.5 million worldwide in 2021, which is approximately one-third of deaths globally [1]. Approximately 80% of these deaths are due to ischemic heart disease and cerebrovascular disease (stroke) [2], which has been estimated to be preventable by focusing on the improvement of risk factors.

Type 1 diabetes mellitus (T1DM) is a common metabolic syndrome characterized by disrupted glucose, fat, and protein metabolism. The prolonged hyperglycemia in T1DM often results in the development of subsequent complications [3]. Several epidemiological studies have established that CVD risk is significantly increased in T1DM patients; men with T1DM have a corresponding 5-fold and women a 10-fold relative risk of developing a cardiovascular event [4]. Moreover, T1DM patients with HbA1c levels less than <6.9% (52 mmol/mol) have a 2-fold increased risk of CVD and mortality compared to controls [5]. There are several suggested mechanisms in which insulin resistance and hyperglycemia in T1DM may lead to vascular complications, many of which involve the activation of nuclear factor κ-B (NFκB), oxidative stress, arterial endothelial cells, vascular smooth muscle cells and macrophages [6]. Previous studies have shown evidence to illustrate that subclinical CVD can be defined as vascular endothelial dysfunction in TIDM [7] as it has been shown to precede the development of atherosclerosis and overt coronary artery disease (CAD) [8]. Subclinical CVD can also be characterized by increased levels of circulating endothelial cells (EC), pro-inflammatory cytokines and reduced levels of circulating endothelial progenitor cells (cEPCs), colony forming units–Hill colonies (CFU–Hill) and pro-angiogenic cells (PACs) [7,9,10,11,12,13].

CFU–Hill colonies have a role in paracrine support of angiogenesis through the release of chemokines, pro-inflammatory cytokines, and growth factors such as interleukin-8 (IL-8), vascular endothelial growth factor (VEGF), and tissue factors [14,15]. The release of these molecules promotes the migration of mature ECs to support local angiogenesis [16] and prevention of oxidative stress-induced apoptosis of mature ECs [17]. Hill et al. investigated the number of CFU–Hill colonies in relation to conventional cardiovascular risk factors and endothelial function in healthy volunteers with no history of cardiovascular disease. They observed that the number of CFU–Hill colonies had an inverse correlation with the Framingham risk score [11]. Similarly, reduced numbers of EPCs predicted poor CVD prognosis and was associated with a significantly higher incidence of cardiovascular events (n = 120, *p* = 0.0009) [18]. Werner reported that in patients with CAD (n = 507), increasing levels of CD34^+^VEGFR-2^+^ EPCs were associated with a lower risk of death from CVD events [19]. Furthermore, studies have shown that reduced CFU–Hill colonies are not only correlated with endothelial function but also with CVD outcomes such as stroke [20,21]. These findings confirm the role of CFU–Hill colonies as prospective biomarkers for early detection and management of CVD; however, further research into CFU–Hill colonies should be explored to provide novel CVD therapies.

Several animal models have shown the role of the dysregulation of various miRNAs in the pathogenesis of many diseases including CVD and therefore may act as sensitive non-invasive biomarkers of CVD. miR-7-5p has been explored and suggested to play a role in many processes resulting in the pathophysiology of CVD. A rat model showed that the overexpression of miR-7-5p resulted in cerebral ischemia/reperfusion in a model of cerebral artery occlusion and consequently miR-7-5p inhibition led to the reversal of occlusion [22]. MiR-7 antagomir experiments promoted angiogenesis by upregulating vascular VEGF while directly targeting Krüppel-like factor 4 (KLF4) [23]. The downregulation of miR-7 promoted tube formation of HUVECs, suggesting miRNA has anti-angiogenic function in endothelial cells. MiR-7 overexpression in transgenic mice has also been shown to result in adverse cardiac dilation, fibrosis, and post-transverse aortic constriction [24]. The overexpression of miR-7-5p has been demonstrated to be anti-angiogenic and makes it an excellent miRNA to further explore the paracrine function of CFU–Hill.

Metformin, an oral biguanide, has been extensively used as a first-line treatment for type 2 diabetes. The UK Prospective Diabetes Study first reported the cardiovascular benefit of metformin in diabetic patients, which reduced the risk of microvascular disease by 25% with a 16% reduced risk of myocardial infarction (MI) (*p* = 0.052, n = 300) [25]. Furthermore, several animal models have illustrated the cardioprotective effects, independent of its antihyperglycemic effect. Experimental evidence suggests that metformin can improve cardiac ischemia–reperfusion injury [26] and hypertension [27] in murine models. Li et al. demonstrated metformin’s anti-atherosclerotic effect by the inhibition of nuclear factor kappa B subunit and the phosphorylation of inhibitor of nuclear factor kappa subunit beta in the vessel wall of experimental atherogenesis of rabbits, and reducing the level of human serum c-reactive protein (CRP) [28]. Furthermore, metformin has been shown to alter the expression of miRNA, downregulating miR-124 expression in type 1 diabetic Apoe−/− mice which increased the stability of the carotid atherosclerotic plaque, reducing overt CVD disease risk [29].

Considering, the anti-angiogenic effect of miR-7-5p demonstrated in animal studies, we therefore hypothesized that the expression of miR-7-5p in CFU–Hill colonies will be upregulated in T1DM patients and downregulated with metformin intervention. We aimed in this study to explore the role of miR-7-5p as a subclinical CVD biomarker and the effect of metformin on miR-7-5p expression in T1DM patients.

## 2. Results

### 2.1. Subjects’ Clinical and Metabolic Characteristics

In this study, 29 T1DM patients and 20 healthy controls (HC) were recruited with matched age and gender. The ages of T1DM patients and HCs are 47.2 ± 12.7 and 46.5 ± 11.7 years (*p* = 0.8), respectively. Patients were well controlled with a mean HbA1c level of 57.3 ± 7.6 mmol/mol with diabetes duration 22.4 ± 13.9 years and no recorded CVD events. The demographic and clinical data of the participants were previously published [30].

### 2.2. Comparisons of Inflammatory and Vascular Health Markers between T1DM and HC

In T1DM patients, the plasma levels of CRP (*p* < 0.001) and IL-10 (*p* = 0.008) were significantly upregulated compared to HC as published before [31]. CFU–Hill colonies (*p* = 0.04), and circulating progenitor cells (CD34^+^/100 lymphocytes *p* < 0.001 and CD34^+^CD133^+^/100 lymphocytes *p* = 0.013) were detected to be significantly lower in T1DM compared to HCs. The effects of metformin on vascular markers were published previously [9].

### 2.3. The Expression of miR-7-5p in T1DM and HC

**Figure 1:**
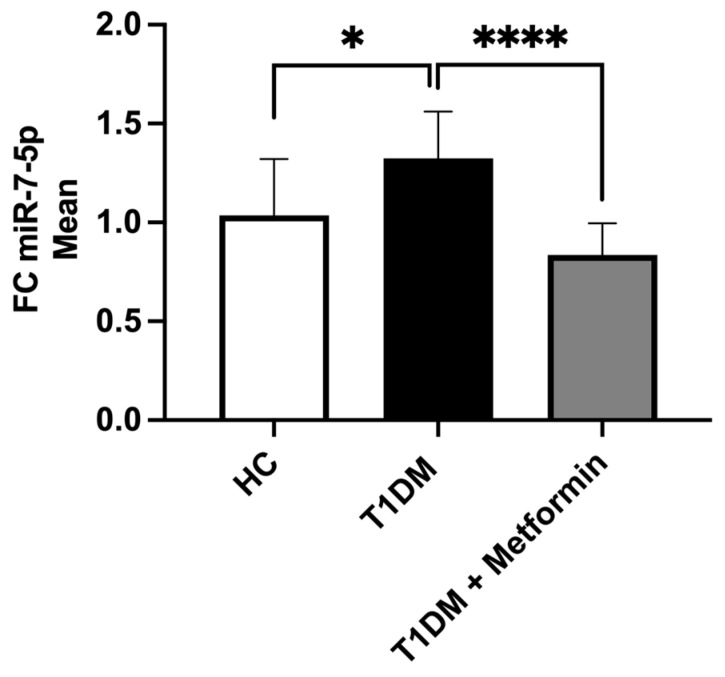
Comparison of miR-7-5p expression in healthy control (HC), type 1 diabetes (T1DM) patients before and after metformin. Data are presented as the means ± SD and the difference between groups was analyzed by an unpaired *t*-test; * *p* < 0.05, **** *p* < 0.0001.

The expression of miR-7-5p in the CFU–Hill colonies was significantly upregulated in T1DM versus HC (*p* = 0.015), whereas no difference in miR-7-5p expression in T1DM versus HC was detected in plasma. After 8 weeks of metformin therapy, there was a significant decrease in miR-7-5p in T1DM patients (*p* < 0.0001), normalizing its expression to those in HCs (Figure 1).

**Figure 2:**
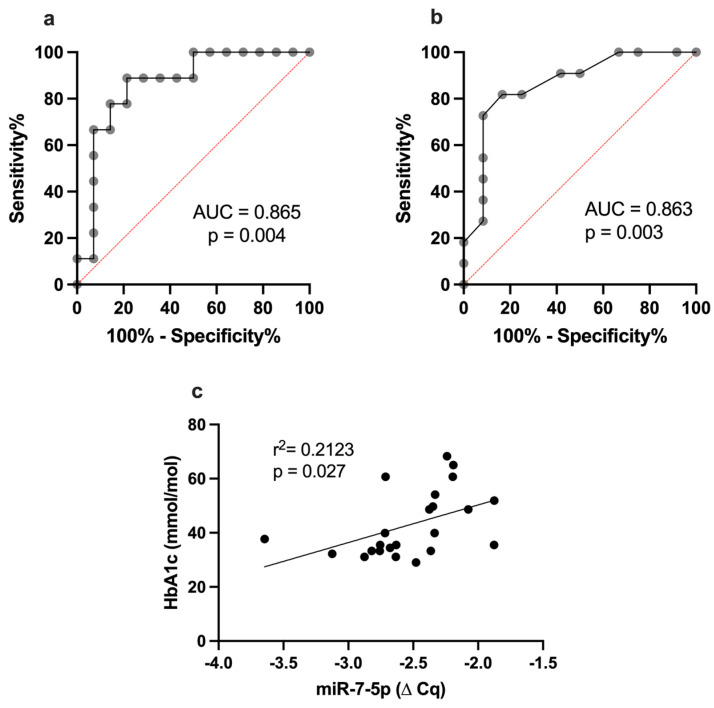
(**a**) ROC curve analysis of miR-7-5p in distinguishing T1DM patients from healthy control; (**b**) ROC curve analysis for HbA1c to define overexpressed miR-7-5p in CFU–Hill colonies; (**c**) Correlation between miR-7-5p and HbA1c using linear regression. AUC: area under the curve; HbA1c: glycated hemoglobin.

Furthermore, receiver operating characteristic (ROC) curve analysis of miR-7-5p reveals the application of this miRNA as a sensitive biomarker for subclinical CVD with an area under the curve (AUC) of 0.8651 (*p* = 0.004), a cut-off value of −2.427, 90% sensitivity and 78.6 specificity (Figure 2a). Additionally, ROC analysis was conducted to determine the cut-off value of HbA1c at which miR-7-5p was upregulated. The HbA1c value was 44.3 mmol/mol (6.2%) with an AUC of 0.863 (*p* = 0.003), 72.73% sensitivity and 91.67% specificity (Figure 2b). A positive correlation was detected between miR-7-5p and HbA1c (*p* = 0.027, Figure 2c).

### 2.4. Correlation of miR-7-5p with Inflammatory Markers

**Figure 3:**
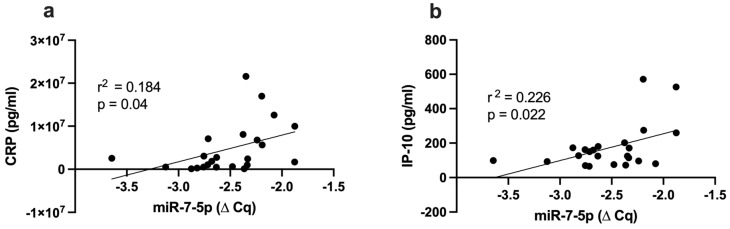
Correlation between miR-7-5p and inflammatory markers (**a**) CRP; (**b**) IP-10. CRP: c-reative protein, IP-10: interferon-**γ**-induced protein 10.

Simple linear regression analysis showed that miR-7-5p was positively correlated with inflammatory marker CRP (r^2^ = 0.184, *p* = 0.04, Figure 3a) and interferon-**γ**-induced protein 10 (IP-10, r^2^ = 0.226, *p* = 0.022, Figure 3b).

### 2.5. miR-7-5p Functional Pathway and Molecular Targets

**Figure 4:**
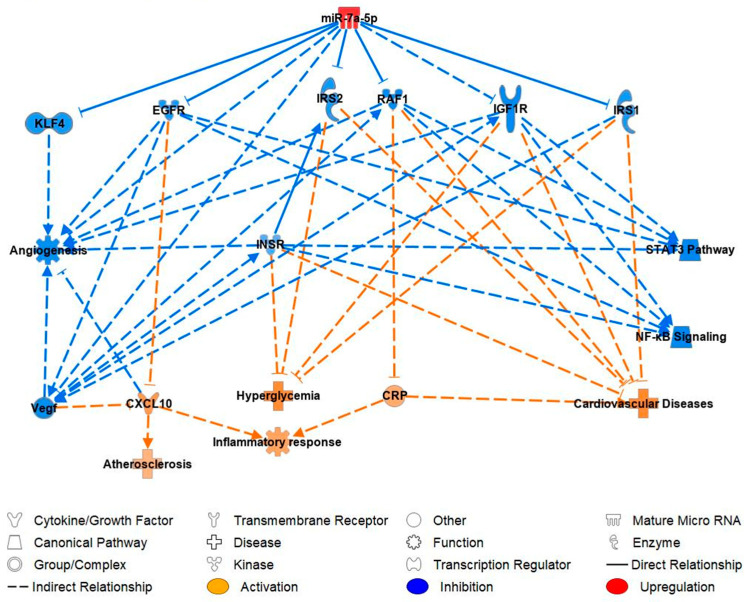
IPA prediction network of miR-7-5p using our data and its mRNA targets and cytokines supporting its contribution to CVD. KLF4: Kruppel-like factor 4; EGFR: epidermal growth factor receptor; IRS1/2: insulin receptor substrate 1/2; RAF1: v-raf- 1 murine leukemia viral oncogene homolog 1; IGF1R: insulin-like growth factor 1 receptor; INSR: insulin receptor; STAT3: signal transducer and activator of transcription 3; NF-κB: nuclear factor kappa-light-chain-enhancer of activated B cells; VEGF: vascular endothelial growth factor; CRP: c-reactive protein; CXCL10: C-X-C motif chemokine ligand 10/IP-10: interferon gamma-induced protein 10.

Ingenuity pathway analysis (IPA) was used to perform the analysis of predicted miR-7-5p-mRNA targets to investigate the miRNA–mRNA relationships in the development of CVD. After inputting miR-7-5p, IP-10 (CXCL10), CRP and glucose into IPA software version 9.0 imitating a diabetic state, it was predicted, through published knock-out studies that miR-7-5p has anti-angiogenic effects and is significantly upregulated in T1DM patients (Figure 4). IPA predicted miR-7-5p activating hyperglycemia, atherosclerosis, CVD, and the inflammatory response, while inhibiting angiogenesis. MiR-7-5p has been proven to inhibit the mRNA expression of KLF4, which results in the inhibition of angiogenesis. It was also proven to inhibit epidermal growth factor receptor (EGFR) which resulted in the activation of atherosclerosis and CXCL10/IP-10 which then further activates atherosclerosis. In addition, miR-7-5p was detected to inhibit insulin-like growth factor 1 receptor (IGF1R), v-raf-1 murine leukemia viral oncogene homolog 1 (RAF1) and insulin receptor substrate 1 (IRS1), which results in the activation of CVD and CRP. The inhibition of insulin receptor substrate 2 (IRS2) and insulin receptor (INSR) was predicted to activate hyperglycemia and CVD (Figure 4).

**Figure 5:**
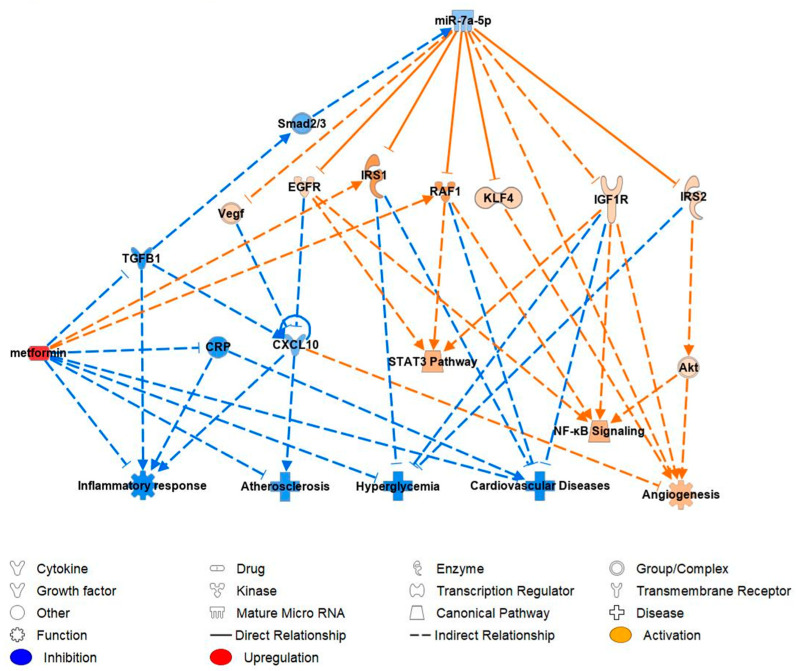
IPA prediction network of miR-7-5p molecular targets and functional pathways following metformin intervention. KLF4: Kruppel-like factor 4; EGFR: epidermal growth factor receptor; IRS1/2: insulin receptor substrate 1/2; RAF1: v-raf-1 murine leukemia viral oncogene homolog 1; IGF1R: insulin-like growth factor 1 receptor; INSR: insulin receptor; STAT3: signal transducer and activator of transcription 3; NF-κB: nuclear factor kappa-light-chain-enhancer of activated B cells; VEGF: vascular endothelial growth factor; CRP: c-reactive protein; CXCL10: C-X-C motif chemokine ligand 10/IP-10: interferon gamma-induced protein 10; TGFβ1: Transforming growth factor beta 1.

Metformin therapy was proven by us to inhibit miR-7-5p expression, which in turn was predicted/proven to activate the mRNA expression of EGFR, RAF1, KFL4, IRS1, IGF1R and IRS2 which were previously downregulated. The effect of metformin on miR-7-5p was proven to occur indirectly via TGFβ1 and Smad2/3. Functional pathway analysis predicted that metformin therapy would inhibit the occurrence of the inflammatory response, atherosclerosis, hyperglycemia and cardiovascular disease and result in the predicted activation of angiogenesis. (Figure 5).

## 3. Discussion

This is the first study investigating the expression of miR-7-5p in CFU–Hill colonies. In our study, we have validated previous animal research showing anti-angiogenic properties of miR-7-5p as we observed upregulation of miR-7-5p expression in CFU–Hill colonies in T1DM patients without overt CVD. MiR-7-5p expression was then downregulated to normal levels after metformin therapy. Additionally, we observed a positive correlation of miR-7-5p with inflammatory markers such as CRP and IP-10 (CXCL-10).

In our study, upregulation of CRP was detected, which had been previously demonstrated to be positively associated with the risk of CVD events. Animal and human studies suggest that CRP may directly contribute to the inflammatory progression of atherosclerosis [32] via reducing the expression of nitric oxide synthase in endothelial cells [33] and significantly increasing expression levels of vascular cell adhesion molecules contributing to the atherosclerotic process [34]. The upregulation of CRP and pro-inflammatory cytokines in these well-controlled T1DM patients further validates the inflammatory state and its potential sequential contribution to the development of CVD, as a model of subclinical CVD.

### 3.1. miR-7-5p Expression in T1DM Patients 

We have shown that miR-7-5p in CFU–Hill colonies is upregulated in T1DM. Our findings are concordant with animal models that show upregulation of miR-7-5p expression [23,24,35]. Functional network analysis of the mouse model of MI and end-stage heart failure (HF) revealed that miR-7 expression was significantly upregulated in ventricular tissues in both model of MI and heart failure (*p* < 0.05) [36]. Additionally, reverse transcriptase-PCR analysis of serum samples confirmed that miR-7-5p was upregulated in left ventricular hypertrophy hypertensive patients compared with healthy subjects [37]. Overexpressed miR-7-5p was found in the serum of type 2 diabetes patients compared to HC [38]. We found the absence of miR-7-5p expression in the plasma of our T1DM patients compared to HC; to our knowledge, no study has measured the miRNA levels in T1DM patients’ plasma.

Furthermore, ROC analysis distinguishes patients with T1DM from HC as it determined the miR-7-5p to define subclinical CVD at a HbA1c level of 44.3 mmol/mol (pre-diabetes) with an AUC value of 0.863, *p* = 0.003. Our study demonstrates the potential of miR-7-5p to be used as a sensitive biomarker for CVD in T1DM. MiR-7-5p was positively correlated with HbA1C (*p* = 0.027), and this may suggest that the regulation of miR-7-5p is intertwined with diabetic control. Latreille et al. demonstrated that transgenic mice overexpressing miR-7 in β cells developed diabetes due to dysfunctional β cell dedifferentiation and insulin secretion [39].

This finding is in line with the prediction shown on IPA analysis showing a positive association between miR-7-5p levels and hyperglycemia. However, as we are the first to study the expression of miR-7-5p in CFU–Hill colonies in T1DM, more studies are required to validate the effect of miR-7-5p on glucose metabolism and insulin sensitivity to confirm our findings.

### 3.2. Positive Association between miR-7-5p and Inflammatory Markers

Our findings showed a positive correlation between miR-7-5p and the levels of C-X-C motif chemokine 10 (CXCL-10) or interferon gamma-induced protein 10 (IP-10). CXCL-10 acts as a chemokine and attracts immune cells to sites of vascular injury, and it has been shown to be expressed in atherosclerotic lesions [40]. In ApoE−/− mice studies, blocking CXCL-10 was shown to inhibit the formation of atherosclerosis [41] and resulted in the development of a more stable plaque phenotype [42]. It has also been reported that serum CXCL-10 was increased in coronary artery occlusion patients [43] and CAD patients [44].

Furthermore, several studies have shown increased serum CXCL-10 levels in T1DM patients compared to non-diabetic patients [45,46]. Our hypothesis that the upregulation of miR-7-5p would be pro-inflammatory was consistent with IPA analysis, which demonstrated that the upregulated miR-7-5p resulted in the activation of CXCL-10 via mRNA epidermal growth factor receptor (EGFR) (Figure 4). EGFR inhibition has been shown to upregulate the expression of pro-inflammatory cytokines such as CXCL-10 [47]. Following metformin treatment, there were significantly reduced levels of CXCL-10 in T1DM. This finding is supported by IPA prediction (Figure 5); metformin’s predicted inhibition of CXCL-10 could potentially result in reduced vascular damage in T1DM patients would ameliorate CVD risk factor development. Targeting EGFR (via activation) may provide a mechanism to manage inflammation in T1DM; however, further research needs to be performed to validate these findings in clinical settings.

Our study showed a positive relationship between miR-7-5p and CRP in TIDM patients. This finding is concordant with prior studies that demonstrate CRP’s significance in chronic inflammatory diseases such as CVD and diabetes mellitus [48]. Additionally, Lu et al. showed that miR-7 was positively correlated with CRP in a model of acute pancreatitis [49]. Several studies showed that CRP is strongly associated with the risk of CVD and ischemia in individuals with no history of vascular disease [48,50]. Even in the absence of CVD, it was found that increased plasma CRP concentrations correlate with endothelial dysfunction markers in T1DM [51]. Additionally, we have confirmed from a previous publication that well-controlled TIDM patients without overt CVD events displayed inflammation via increased pro-inflammatory cytokines [52]. CRP has been demonstrated to engage in thrombus formation and significant expression of adhesion molecules in endothelial cells, therefore contributing to the development of atherosclerosis [53]. IPA findings supported our hypothesis demonstrating the predicted activation of inflammatory response. The increase in CRP and IP-10 in these patients further validates T1DM as a chronic inflammatory state that is characterized by vascular and endothelial dysfunction as a major contribution to the development of CVD.

### 3.3. Downregulation of miR-7-5p by Metformin

This is the first study that has investigated the effect of metformin on miR-7-5p in CFU–Hill colonies. Currently, metformin is extensively used as a first-line treatment for type 2 diabetes mellitus (T2DM) due to its efficacy in blood glucose control [25,54]. We have previously published a study regarding the potential cardioprotective effect of metformin in T1DM patients via the improvement of cEPCs, cECs, CFU–Hill colonies and PAC count and adhesion [9]. In this present study, following metformin therapy, we observed a downregulation of miR-7-5p expression in comparison to its expression level before metformin therapy.

Our results are concordant with animal studies as several animal models have illustrated the cardioprotective effect of metformin therapy on miRNA expression, independent of its antihyperglycemic effect. Metformin has been shown to improve ischemia–reperfusion injury and hypertension in murine models [26,27] and significantly reduced left ventricular mass, systolic blood pressure and oxidative stress, which are also CVD risk factors in the MET- REMODEL clinical trial [55].

Our findings established the evidence to support metformin intervention as a cardioprotective drug. The inhibition of miR-7-5p was proven by IPA to occur via TGFβ1 and Smad2/3 protein. TGF-β1 is a multifunctional cytokine, that has been associated with ventricular remodeling, cardiac fibrosis and hypertrophy [56]. Blocking TGF-β1 had been previously shown to prevent myocardial fibrosis [57]. Previous studies have demonstrated that metformin reduced cardiac fibrosis by inhibiting TGF-β1 and Smad3 pathway [58]. Fei et al., also found that metformin inhibits the expression of fibrosis biomarkers in EPCs [59]. Thus, metformin has the potential to improve EPC function in CVD. We are the first group to report the predicted mechanism of how metformin reverses the cardio-detrimental effects of miR-7-5p via activation of target genes which are involved in insulin signaling, inflammation, fibrosis, and cardiac hypertrophy. This can be mediated through improvement in inflammatory state, EPC mobilization and survival.

### 3.4. Ingenuity Prediction Model: Pathway Analysis of miR-7-5p in Reference to Cardiovascular Function

IPA predicted that upregulation of miR-7-5p would directly inhibit KLF4, EGFR, IRS1/2, and RAF1 and indirectly IGF1R. The inhibition of these mRNAs was predicted to subsequently activate the expression of CXCL10/1P-10 and CRP. This finding supports our miRNA–mRNA correlation analysis, which also showed that the upregulation of miR-7-5p was positively correlated with IP-10 and CRP levels. Additionally, miR-7-5p upregulation was found to be cardio-adverse as it was predicted to activate inflammatory response, atherosclerosis, hyperglycemia, cardiovascular disease as well as inhibit angiogenesis and STAT3 and NF-KB pathway. Consequently, downregulating the expression of miR-7-5p may play an important role in future therapies to prevent and manage CVD. Treatment with metformin was shown to be cardioprotective. The cardioprotective mechanism of metformin is illustrated by the proven inhibition of TGFB1 which simultaneously inhibits Smad2/3. In addition, the action of metformin results in the predicted activation of KLF4, EGFR, IRS1/2, RAF1, IGF1R, also including vegf and angiogenesis.

### 3.5. Clinical Applications for Future CVD Research

This research has identified potential miRNA-based therapies for the management of CVD. The novel discovery of downstream target genes involved in CVD development may be used for future targeted therapy for vascular diseases. Incidentally, our target genes have already been explored in CVD research, so our research is concordant with ongoing research into CVD management therapies.

#### 3.5.1. Epidermal Growth Factor Receptor (EGFR)

EGFR (also known as ErbB1) is a transmembrane protein tyrosine kinase receptor expressed on endothelial cells, monocytes, macrophages and is a member of the ErbB family of receptor tyrosine kinases [60]. EGFR is proangiogenic as, when overexpressed, results in cell proliferation, migration, inflammation, and differentiation [61]. Knock-out experiments of the EGFR gene led to cardiac hypertrophy with fibrosis and inflammation [62]. Recent studies showed signaling through EGFR (activation) was shown to provide cardioprotection against stress-induced injury [63]. In animal models, the inhibition of EGFR resulted in significant changes to the left ventricular wall thickness and altered cardiovascular function in in vivo mouse models [64]. miR-7 has been shown to directly inhibit EGFR expression [65].

#### 3.5.2. Insulin Receptor Substrate 1 and 2 (IRS1/IRS2)

Insulin receptor substrate 1 (IRS1) is a gene that encodes a 131 kDa protein, phosphorylated by insulin receptor tyrosine kinase. Phosphorylation of IRS-1 and -2 tyrosine activates IRS proteins to bind to PI3K and PKB/Akt signaling molecules in the insulin signaling pathway [66]. Double-knock-out studies have shown that cardiac suppression of IRS1 and IRS2 increases apoptosis, results in cardiac dilation and heart failure in neonatal rat ventricular cardiomyocytes [67]. In another knock-out study, IRS1 deletion resulted in accelerated atherosclerosis in Apoe−/−-knock-out mice by increasing vascular dysfunction and inflammation [68]. IRS1 was found to protect against endothelial cell injury of ox-LDL- induced by activating the Akt/FoxO1 signaling pathway and inhibiting ER stress/oxidative stress-mediated apoptosis [69], highlighting the importance of IRS1 in CVD development. An IRS-2−/− deficient mice model showed progressive neointima formation in response to vessel injury, additionally these mice exhibited increased FFA, triglyceride levels and hypertension [70]. Cao et al. found that the overexpression of miR-7 downregulated IRS-1 in a diabetic rat model [35].

#### 3.5.3. Raf-1 Proto-Oncogene (RAF1)

RAF1 or c-RAF is a family of serine/threonine kinases expressed in the heart. Raf proteins activate the conserved MAPK/extracellular signal-regulated (ERK) signaling pathway cascade that plays a role in cell survival, proliferation, and migration [71]. Harris et al. reported a transgenic study using dominant-negative Raf to inhibit the ERK pathway in the heart and demonstrated attenuated hypertrophy and gene induction during pressure overload [72]. Furthermore, Yamaguchi et al. described a cardiomyocyte-specific c-Raf-1 knock-out mice model demonstrated, the mice developed heart failure and a significantly increased number of apoptotic cardiomyocytes without hypertrophy [73]. Overexpression studies demonstrate that miR-7-5p significantly reduces RAF-1 mRNA and protein expression in human umbilical vein endothelial cells [74], which supports this study’s findings.

#### 3.5.4. Insulin-like Growth Factor 1 Receptor (IGF-1R)

IGF-1R is a polypeptide hormone receptor involved in the regulation of autophagy, hypertrophy, metabolism, and apoptosis in the heart IGF-1 is produced and released by cardiac fibroblasts and promotes cardiomyocyte hypertrophy via paracrine signaling [75]. Tejada et al. found that the inhibition of IGF-1R in post-ischemia–reperfusion-injured mice exacerbated cardiac left ventricular systolic function [76]. Knock-out of IGF-1R in cardiac muscle increased early-onset dilated cardiomyopathy and heart failure in mice [77]. miR-7 overexpression has been shown to inhibit IGF-1R in human squamous cells [78].

#### 3.5.5. Kruppel-like Factors-4 (KLF4)

KLF4 is a transcription factor that regulates cardiac macrophage proliferation, angiogenesis, and endothelial inflammation. Knock-out in myeloid cells from mouse decreases angiogenesis in mouse heart that involves pressure overload hypertrophy in mouse [79]. Another knock- out study showed that cardiac-specific deletion of the Klf4 gene significantly increased mice susceptibility to heart failure and death following pressure overload induced by TAC [80]. miR-7 has been shown to directly inhibit KLF4 IN HUVECs, supporting our findings [23].

### 3.6. Contribution/Causation

Based on our correlation analysis, we have identified possible contribution or causality generated from miR-7-5p target genes binding sites (Table 1). Utilizing the TargetScan Human, release 7.1 and Diana-TarBase v8 databases, we explored probable target genes of interest for miR-7-5p. Moreover, the canonical pathway tool in IPA was used to establish the most relevant canonical pathways associated with the target genes. MiR-7-5p has effects on multiple pathways in IPA, of which many are interrelated. MiR-7-5p has binding sites in 6 target genes of interest. It was observed that the highest number of binding sites was present among the STAT3 pathway.

It is evident that miR-7-5p has a causal role via the STAT3 pathway, in which molecules including VEGF, EGFR, INSR, RAF1 and IGF1R were found to be a part of this pathway. This pathway is predicted to be involved in the development of atherosclerosis and hyperglycemia. The NF-kB signaling pathway is also observed with molecules EGFR, INSR, RAF1 and IGF1R involved in the pathway, this pathway is involved in cardiovascular disease and angiogenesis. The next causal effect is KLF4, which is involved in angiogenesis. Another observed causal affect is via IRS1/2 which are involved in the development of hyperglycemia and cardiovascular disease. miR-7-5p is also observed to have a casual effect on EGFR, which is involved in the development of atherosclerosis and inflammatory response.

## 4. Materials and Methods

### 4.1. Study

In this cross-sectional study, T1DM patients with good glycemic control (HbA1c 7.4 ± 0.7% [57.3 ± 7.6 mmol/mmol]), free from diabetic complications (active proliferative retinopathy, stage 3b renal impairment, i.e., eGFR < 45 mL/min/1.73 m^2^) or macrovascular disease, were recruited. There were 29 T1DM patients and 20 non-diabetic HCs matched for age (47.2 ± 12.7 years, 46.5 ± 11.7 years, respectively, *p* = 0.8) and sex. Blood samples were obtained after an overnight fast.

This study was carried out in line with the Helsinki Declaration. T1DM subjects were recruited from either Queen Elizabeth Hospital, Gateshead or Royal Victoria Infirmary, Newcastle, United Kingdom. Approval from NHS Health Research Authority, NRES Committee Northeast—Sunderland, United Kingdom (Research Ethics Committee Reference Number 12/NE/0044,) as well as informed consent from all subjects were obtained.

### 4.2. Cytokine Analysis

K15050D V–PLEX Cytokine Panel 1 human kit, K15049D V–PLEX Pro–inflammatory Panel 1 human kit, K15190D V–PLEX Angiogenesis Panel 1 human kit and K151JFC human TIMP–1 kit (Meso Scale Discovery, Rockville, MD, USA) were used to assay interleukin 7 and 8 (IL–7, IL–8), vascular endothelial growth factor C (VEGF–C), tumor necrosis factor alpha (TNF–α) and circulating vascular cell adhesion molecule 1 (sVCAM–1) in plasma samples from patients and HCs according to the manufacturer’s protocol. Meso Scale Discovery Sector Imager 2400 was used to read plates while Meso Scale Discovery Workbench 2.0 software was used to analyze the data.

### 4.3. Evaluation of Circulating Endothelial Progenitor Cells by Flow Cytometry

cEPCs were defined as CD45^dim^CD34^+^VEGFR–2^+^ cells and cECs as CD45^dim^CD133^−^ CD34^+^ events were measured by flow cytometry on a BD FACS Canto^TM^ II system (BD bioscience, San Jose, CA, USA) as described previously [5]. Enumeration of proangiogenic cells (PACs) in vitro assay was previously described by us [5].

### 4.4. Culture and Quantification of CFU–Hill Colonies

CFU–Hill colonies were cultured according to the method described previously [31].

### 4.5. Plasma and Hill Colonies miRNA Extraction

Blood samples were centrifuged at 500× *g* for 15 min. The platelet-rich plasma (upper fraction of the centrifuge) was then re-centrifuged at 13,000× *g* for another 5 min to obtain platelet-free plasma, which then was stored at −80 °C to be analyzed later. Hemolysis was checked for all samples. To monitor hemolysis two microRNAs are used; one that is expressed in red blood cells (miRNA-451) and one that is relatively stable in serum and plasma and not affected by hemolysis (miRNA-23a). Samples with ratios above 7.0 have an increased risk of being affected by hemolysis [3]. An aliquot of 200 μL per sample was transferred to a FluidX tube and 60 μL of Lysis solution BF containing 1 μg carrier–RNA per 60 μL Lysis Solution BF and RNA spike-in template mixture was added to the sample and mixed for 1 min and incubated for 7 min at room temperature, followed by addition of 20 μL Protein Precipitation solution BF. Total RNA was extracted from the samples using miRCURY RNA isolation Kit-Biofluids; high-throughput bead-based protocol (Exiqon, Vedbaek, Denmark) in an automated 96-well format. The purified total RNA was eluted in a final volume of 50 μL. Plasma miRNAs were isolated from plasma samples using RNA isolation protocol optimized for plasma by QIAGEN (Exiqon Services, Denmark). Agilent 2100 (Santa Clara, CA, USA) was used to assess the RNA samples’ integrity, giving high RNA Integrity Numbers (RIN) that ranged from 9.1 to 10.

Total RNA was extracted from Hill colonies using miRNeasy Micro Kit (QIAGEN, Hilden, Germany). The cell pellet was lysed in 700 µL QIAzol and 1.25 µL of each: UniSpike 2, 4, 5 and MS2 carrier RNA (Roche, Basel, Switzerland) was directly added to the QIAzol lysate. After that, the sample was vortexed and kept in the dark for 5 min. DNase on-column treatment (QIAGEN, Hilden, Germany) was performed according to the manufacturer’s instructions.

### 4.6. Quantitative Real–Time Polymerase Chain Reaction for miRNA

miRCURY LNA RT Kit (QIAGEN, Hilden, Germany) was used to assay miRNA quantitative real–time polymerase chain reaction (qRT–PCR) in plasma and Hill colonies as described previously in detail [31]. Briefly, cDNA was diluted 100× and assayed in 10 μL PCR reactions according to the protocol for miRCURY LNA miRNA PCR; miRNA was assayed once by qPCR on the miRNA Ready-to-Use PCR, Human panel I + II (Catalog number: 339322, QIAGEN) using miRCURY LNA SYBR Green master mix. The amplification was performed in a LightCyclerR 480 Real-Time PCR System (Roche, Basel, Switzerland) in 384 well plates and analyzed using the Roche LC software 4 (Basel, Switzerland) both for the determination of Cq. All Cq data were normalized using the global mean method based on the average of the assays detected in all samples, yielding ΔCq values. Fold-change analysis was performed using 2 × |ΔΔCq| calculation, with ΔΔCq obtained from (∆Cq × T1DM) − (∆Cq × HCs).

### 4.7. Ingenuity Pathway Analysis (IPA) of miR-7-5p

The functional pathways, cellular functions, and target genes regulated by miR-7-5p were identified using IPA software version 9.0 (Ingenuity, Redwood City, CA, USA).

The interaction site prediction between the transcripts and miR-7-5p was performed using TargetScan Human, release 7.1 (www.targetscan.org, accessed on 13 April 2023), and Diana–TarBase v8, (http://carolina.imis.athena–innovation.gr/diana_tools/web/index.php?r=tarbasev8%2Findex, accessed on 13 April 2023) [6] databases.

### 4.8. Statistical Analyses

All data were presented as the mean ± standard deviation, unless otherwise stated. The Shapiro–Wilk test was used to assess the normality of the data. The significance of the data was assessed using an unpaired *t*-test or the Mann–Whitney test. The correlation between miR-7-5p expression and other markers was measured using linear regression tests. ROC curve analysis of miR-7-5p in study participants was carried out to assess the sensitivity of miR-7-5p as a biomarker for subclinical CVD in T1DM. Moreover, the ROC curve was used to determine the cut-off value for miR-7-5p upregulation. Statistical analysis was performed using GraphPad Prism 9.0 (GraphPad software, San Diego, CA, USA). A *p* < 0.05 was considered statistically significant.

## 5. Conclusions

Our study has validated animal and in vivo research on anti-angiogenic miR-7-5p as we have observed significant upregulation of miR-7-5p in CFU–Hill colonies in T1DM patients. Moreover, our findings illustrate the cardioprotective mechanism of metformin therapy and suggest that miR-7-5p may be used as a sensitive and non-invasive biomarker for CVD management in the clinic. The methodological limitations of our investigation prevent us from collecting enough RNA from CFU–Hill colonies to evaluate miRNA and mRNA simultaneously in each subject without pooling RNA samples from patients and HCs. This is the first study ever conducted in miR-7-5p in CFU–Hill colonies and should be viewed as a pilot/feasibility study giving key building blocks for future research, despite the other restriction being the relatively small number of participants investigated. Further large-scale studies are however needed to validate the effect of miR-7-5p expression in T1DM patients in clinical settings and its potential applications as a prognostic biomarker in T1DM for CVD monitoring and management.

## Figures and Tables

**Table 1:** Predicted consequential pairing of target genes in the transcript and miR-7-5p.

Target Gene	Representative Transcript	Gene Name	Transcript Position	Predicted Consequential Pairing of Target Region. Transcript (Top) and miRNA (Bottom)	Site Type
KLF4	ENST00000374672. 4	Kruppel-like Factor 4	66–72 3′ UTR	(Transcript) 5′…UUUACUUUUCACACUGUCUUCCC… (miRNA) 3′UGUUGUUUUAGUGAU–**CAGAAGG**U	7mer– m8
			574–580 3′ UTR	(Transcript) 5′…GGAAAAUCUAUAUUU**GUCUUCC** G… (miRNA) 3′ UGUUGUUUUAGUGAU–––**CAGAAGG**U	7mer– m8
IRS1	ENST00000305123. 5	Insulin Receptor Substrate 1	908–915 3′ UTR	(Transcript) 5′ …GAAGAGGAAAUUAAA– **GUCUUCCA**… (miRNA) 3′ UGUUGUUUUAGUGAU– **CAGAAGG**U	8mer
IRS2	ENST00000375856. 3	Insulin Receptor Substrate 2	1588–1594 3′ UTR	(Transcript) 5′…AAUGGCAAUGCAAAA**GUCUUCC**U.. (miRNA) 3′ UGUUGUUUUAGUGAU– **CAGAAGG**U	7mer– m8
			2307–2314 3′ UTR	(Transcript) 5′…AACUUAUCUUGCUCU**GUCUUCCA**.. (miRNA) 3′UGUUGUUUUAGUGAU–**CAGAAGG**U	8mer
EGFR	ENST00000275493. 2	Epidermal Growth Factor Receptor	457–464 3′ UTR	(Transcript) 5′…GAGCACAAGCCACAA**GUCUUCCA**… (miRNA) 3′UGUUGUUUUAGUGAU–**CAGAAGG**U	8mer
RAF1	ENST00000251849. 4	Raf-1 Proto–Oncogene, Serine/ Threonine Kinase	674–681 3′ UTR	(Transcript) 5′ …AUCAUGCUGAAUUUU––**GUCUUCCA**… (miRNA) 3′ UGUUGUUUUAGUGAU– **CAGAAGG**U	8mer
IGF1R	ENST00000268035. 6	Insulin-Like Growth Factor 1 Receptor	5950–5957 3′ UTR	(Transcript) 5′…AUCUUCAGUAUCUUG**GUCUUCCA**… (miRNA) 3′UGUUGUUUUAGUGAU**CAGAAGG**U	8mer

The databases, TargetScan Human, release 7.1 (www.targetscan.org, accessed on 13 April 2023) and Diana–TarBase v8 (http://carolina.imis.athena–innovation.gr/diana_tools/web/index.php?r=tarbasev8%2Findex, accessed on 13 April 2023) were used to predict the sites of interaction between miR-7-5p and the gene transcripts. Predicted accompanying joining of target region transcript and miRNA were highlighted in bold.

## Data Availability

Not applicable.
